# Effect of Filling Material Properties on 1-3 Piezoelectric Composite Performance

**DOI:** 10.3390/mi15070812

**Published:** 2024-06-22

**Authors:** Yao Liu, Yang Zhou, Zhigang Zhao, Jinjie Zhou

**Affiliations:** Shanxi Key Laboratory of Advanced Manufacturing Technology, North University of China, Taiyuan 030051, China; liuyao@nuc.edu.cn (Y.L.); zhou13294601507@163.com (Y.Z.); zhao105820@163.com (Z.Z.)

**Keywords:** piezoelectric composite, vibration coupling, acoustic impedance, filling epoxy mixture

## Abstract

The 1-3 piezoelectric composite is the key component of the acoustic transducer, which is widely used in detection, due to the high energy conversion efficiency, cheap raw material, and low aging. To reveal the influence of epoxy mixture, used to connect the piezoelectric column, on the composite performance, a 1-3 piezoelectric composite model was built. The effects of mixture properties on the impedance curves, vibration mode, and deformation displacement of the composite were determined. Six 1-3 piezoelectric composites with different filling mixture properties, by changing the glass microspheres’ mass ratio in the epoxy, were prepared and measured to validate the model. The results showed that with the increase in the proportion of the glass microsphere in the epoxy mixture, the vibration coupling of the piezoelectric composites was gradually eliminated. The acoustic impedance was reduced by 12%. The electromechanical coupling coefficient and effective electromechanical coupling coefficient were increased by 5.4% and 8.3%, respectively. The density and Young’s modulus decrease in filling mixture can significantly improve piezoelectric composite performance.

## 1. Introduction

Piezoelectric composites have been widely used in medical ultrasound imaging and non-destructive testing [1] as the energy convention component of transducers [2,3], which detect abnormal human tissues and small defects in semiconductor component. The 1-3 piezoelectric composite often uses epoxy to connect the piezoelectric column array [4], which can effectively reduce the acoustic impedance, increase transmission sensitivity, improve resolution, increase energy conversion efficiency, and increase flexibility [5,6,7,8]. However, when the periodic piezoelectric composite was excited by an electrical signal at a certain frequency, the vibrations caused the piezoelectric columns to deform and generate the acoustic wave in the longitudinal and lateral direction, which was easy to produce Bragg diffraction [9]. The irregular arrangement of the PZT ceramic columns increases the machining demand and accuracy, which increases the fabrication cost. So, the change in the filling material and properties becomes the most cost-effective method to improve the 1-3 piezoelectric composite’s performance.

Research on the filling material effect on 1-3 piezoelectric composite performance has been conducted. Liu et al. [10] selected three thermoplastic epoxies to prepare PZT/epoxy piezoelectric composites by the hot pressing process and found that the epoxy has a great influence on the piezoelectric, dielectric, ferroelectric, and acoustic properties of the composite. Huang et al. [11] indicated that the thickness vibration can be significantly enhanced when the impedance was close to concrete by preparing a 1-3 cement-based composite based on epoxy-modified sulfoaluminate cement. Li et al. [12] prepared a high-temperature-resistant 1-3 composite transducer using organic cyanate as the filling material, which could work at about 300 °C compared with traditional 1-3 epoxy composites and had the characteristics of low dielectric loss and high thickness vibration coupling. He et al. [13] used 3D printing technology to prepare an air-based 1-3 piezoelectric composite transducer for defect detection and achieved good results. Zhong et al. [14] designed a 1-3 double-layer epoxy piezoelectric composite based on the series-parallel structure and acoustic matching characteristics and prepared a curved surface transducer to increase the electromechanical coupling coefficient. Wang et al. [15] proposed a three-phase material modified 1-3 piezoelectric composite, which effectively reduced the vibration coupling of the 1-3 piezoelectric composite. Zhao et al. [16] used the finite element method to verify the effects of epoxy elastic modulus and Poisson’s ratio on the series resonance frequency, parallel resonance frequency, and electromechanical coupling coefficient of 1-3 piezoelectric ceramic composites. Wang et al. [17] prepared three different epoxies-based piezoelectric fiber composites and tested the free strain performance o at an ambient temperature of −15~60 °C. However, the mechanism of the filling material effect on the 1-3 piezoelectric composite performances, including the vibration coupling, is not clear now. 

In this study, a model was built in COMSOL (https://cn.comsol.com/) to study the filling material properties and thickness effects on 1-3 piezoelectric composite performance. A six 1-3 piezoelectric composites were prepared by the dicing and filling method with different filling mixture properties by changing the glass microsphere mass ratios, which was used to validate the simulation model. A conclusion was given at last.

## 2. Simulation Model and Experiment Setup

### 2.1. Simulation Model

**Figure 1:**
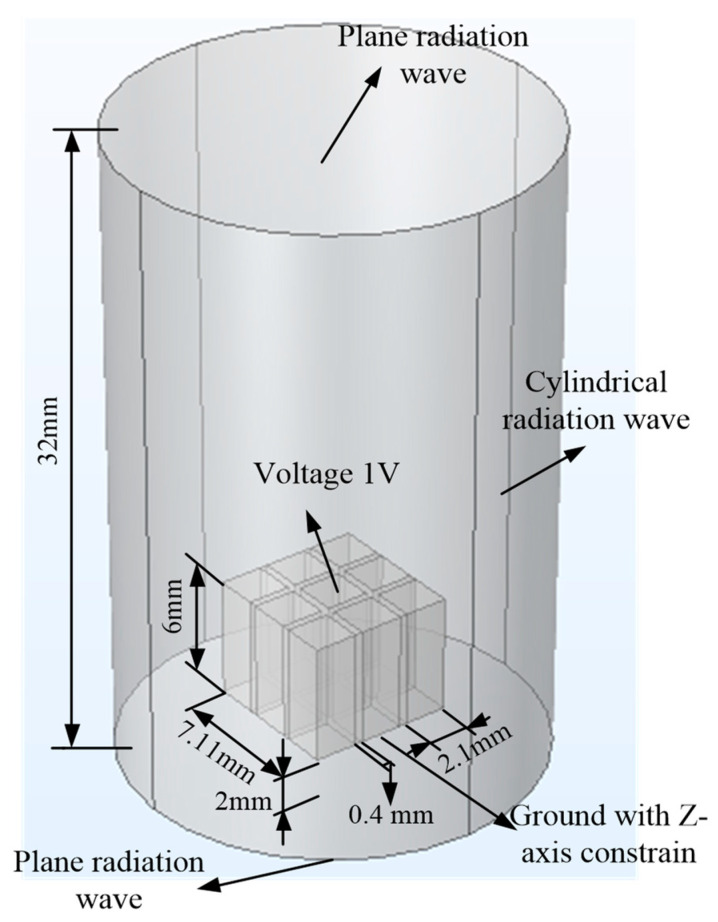
Simulation modeling.

Figure 1 gives the simulation model for the 1-3 piezoelectric composite, which consists of air and the 1-3 piezoelectric composite. The 1-3 piezoelectric composite were combined with PZT-4 columns and epoxy. To save computational time, the 1-3 piezoelectric composite model was built with 3 × 3 columns, which have the same shape and size as 1-3 piezoelectric composite material prepared in experiments later. The air domain was a cylinder with a radius of 10 mm and a height of 32 mm, which was used for acoustic wave propagation. The piezoelectric composite model was placed in the middle of the air domain with 2 mm from the bottom surface. The properties of the piezoelectric composite and air are given in Table 1.

The simulation was carried out through the multiple physical fields in COMSOL. Two types of constitutive equations were used, which were stress charge type and strain charge type. This simulation uses a stress charge pattern as follows:

(1) $$\left\{\begin{array}{l} T=c_{E}S-e^{T}E\\ D=eS+\varepsilon_{S}E \end{array}\right.$$

where *T* is stress, *S* is strain, *E* is the electric field, *D* is potential shift, *c_E_* is the elastic matrix, *e* is the coupling matrix, and *Ɛ_S_* is the dielectric matrix.

The elastic matrix of piezoelectric ceramics is as follows:

(2) $$c_{E}=\left[\begin{array}{cccccc} 138.999 & 77.8366 & 74.2836 & 0 & 0 & 0\\ 77.8366 & 138.999 & 74.2836 & 0 & 0 & 0\\ 74.2836 & 74.2836 & 115.412 & 0 & 0 & 0\\ 0 & 0 & 0 & 25.641 & 0 & 0\\ 0 & 0 & 0 & 0 & 25.641 & 0\\ 0 & 0 & 0 & 0 & 0 & 30.581 \end{array}\right]\mathrm{GPa}$$

The coupling matrix of piezoelectric ceramics is as follows:

(3) $$e=\left[\begin{array}{cccccc} 0 & 0 & 0 & 0 & 12.7179 & 0\\ 0 & 0 & 0 & 12.7179 & 0 & 0\\ -5.20279 & -5.20279 & 15.0804 & 0 & 0 & 0 \end{array}\right]{C/m}^{2}$$

The relative dielectric constant matrix is as follows:

(4) $$\varepsilon_{S}=\left[\begin{array}{ccc} 762.5 & 0 & 0\\ 0 & 762.5 & 0\\ 0 & 0 & 663.2 \end{array}\right]$$

Through exciting the vibration of the piezoelectric ceramic, the acoustic wave generates and propagates into the air. The stress field and the sound pressure field distribution occur in the composite and air, respectively. Therefore, pressure acoustics also need to be incorporated in the simulation.

To observe the sound field when the vibration reaches a steady state, frequency domain simulations were conducted. In the simulation of the sound field, to simplify the calculation process, three assumptions were made. Firstly, perturbation was assumed. Secondly, no liquidity was in the field. Thirdly, no viscosity and heat loss were assumed. Based on those three assumptions, the wave equation of pressure acoustics in the frequency domain could be obtained as follows:

(5) $$\nabla \cdot \left(-\frac{1}{\rho_{0}}(\nabla p-q)\right)-\frac{\omega^{2}p}{\rho_{0}c^{2}}=Q,$$

where *ρ*₀ is the density of the fluid medium, ∇p is the sound pressure, *q* is the dipole source, *ω* is the angular frequency, *c* is the sound velocity in the fluid medium, and *Q* is the monopole source.

This simulation requires the acoustic structural boundary and piezoelectric effect to be conducted simultaneously. For air, a pressure acoustic frequency domain physical field is required. As the air domain in the model was limited, the cylindrical wave radiation boundary and plane wave radiation boundary were added to the air domain surface to prevent sound wave reflection. For piezoelectric ceramics and epoxy mixture, solid mechanics physical fields were required. The piezoelectric material model and linear elastic material model were used to describe the piezoelectric ceramics and epoxy mixture properties, respectively. To fix the bottom surface of the piezoelectric composite, roller support was added. Afterward, the electrostatic physical field and fixture constrain were applied to the bottom surfaces of all piezoelectric columns. The terminal boundaries were added to the upper surfaces of all piezoelectric columns with a voltage of 1 V. To discover the sound field variation caused by the different filling material, ultrasound wavelength should be analyzed. The grid size in the air was set to less than 1/5 of the acoustic wavelength to generate a better transition and made the simulation more realistic.

In the simulation, the scanning frequency range was 50–250 kHz with a coarse step of 5 kHz. Among the specific frequency ranges, such as the coupling frequency, resonant frequency, and anti-resonant frequency, a fine step of 1 kHz was used to scan again. The density, Young’s modulus, and Poisson’s ratio of epoxy mixture with different glass microsphere mass ratios in piezoelectric composites were measured, as given in Table 2. The specific parameters of the glass microsphere used in this study are shown in Table 3.

### 2.2. Improved Dicing and Filling Method

**Figure 2:**
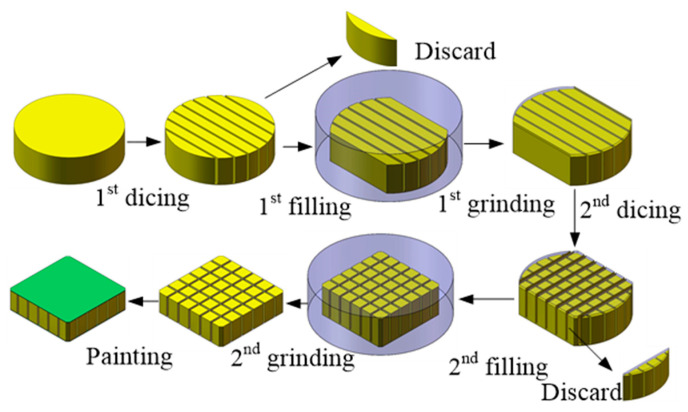
Improved dicing and filling method.

Based on the fabrication experience of the 1-3 piezoelectric composite, an improved dicing and filling method was proposed, shown in Figure 2. Since ceramics are hard and brittle materials [18,19], damages are to generate during the dicing process. This dicing and filling method can effectively reduce the collapse of ceramic columns. The 1st dicing on PZT-4 was conducted to form the kerfs in one direction. To keep the relative position of the kerfs and PZT sheets, the dicing depth should be lower than the thickness to reserve part of the material on the bottom. Then, the PZT sheets on both sides were removed, and the kerfs were filled with the epoxy mixture. The composite was put in the vacuum oven for curing and bubble release. Excess epoxy on the surface was removed by grinding. The composite rotated 90° compared to the 1st dicing direction to conduct the 2nd dicing and filling. The 2nd grinding was used to remove the excess epoxy and uncut layers on the bottom to obtain the specific thickness based on the frequency needed. Finally, conductive metal was painted on the upper and lower surfaces of the piezoelectric composite to form electrodes.

### 2.3. Experiment Setup

**Figure 3:**
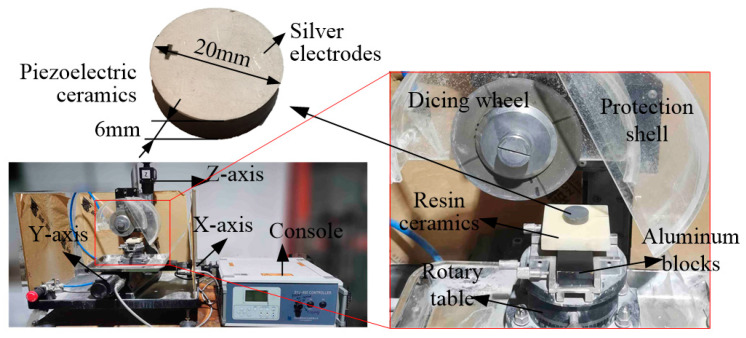
Dicing setup and piezoelectric ceramic.

The dicing was conducted on SYJ-400CNC (Shenyang Kejing Auto-instrument Co., Ltd., Shenyang, China), shown in Figure 3. A console was used to control the moving of the X-, Y-, and Z-axis. The dicing wheel was mounted on the Z-axis. The workpiece was put on the rotational fixture of the XY table. This fixture can rotate at a design angle to adjust the dicing direction. The piezoelectric ceramic was bonded to a white ceramic block by wax, which was clamped to the XY table. During the dicing process, the dicing wheel was moved to specify depth along the Z-axis. Then, the XY table took the workpiece to feed in the X- and Y-axis direction.

The PZT-4 ceramic (Dongguan Xizhe Electronics Co., Ltd., Dongguan, China) used in this study is shown in Figure 3, which has a diameter of 20 mm and 6 mm thickness. The sintered diamond dicing wheel has a diameter of 100 mm and 0.33 mm thickness (Shenyang Kejing Automation Instrument Co., Ltd., Shenzhen, China), which can generate a 0.4 mm width kerf due to the wheel vibration. The grain size of the diamond is 30~40 µm. Table 4 gives the dicing parameters, which were optimized in our previous study [20]. The wheel speed is 2500 rpm, and the feed speed is 5 mm/min. The spacing between the kerfs is 2.5 mm. The depth of the dice is 5.8 mm to keep the 0.2 mm layer uncut. During the dicing process, water is used for cooling, with a flow rate of 720 mL/min. The vacuum in the oven (Dongguan Qinzhuo Environmental Testing Equipment Co., Ltd., Dongguan, China) is at −0.1 MPa for half an hour with 50 °C. The curing continues under atmospheric conditions for 12 h.

**Figure 4:**
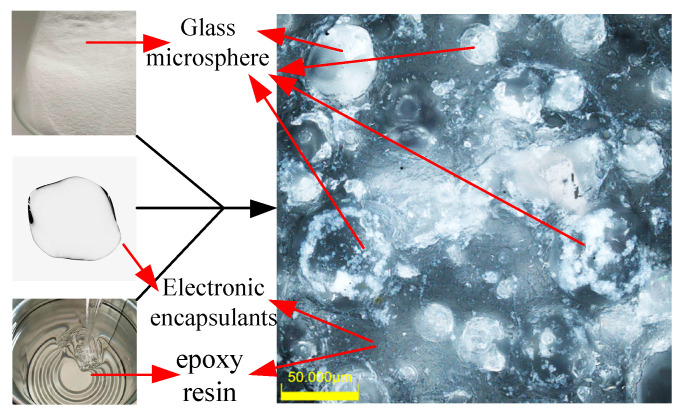
Micrograph of epoxy mixture.

**Figure 5:**
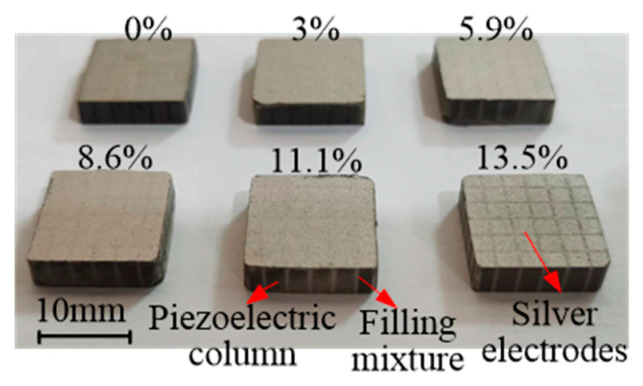
Piezoelectric composite.

To study the influence of filling material properties on piezoelectric composite performance, the mixture of epoxy resin and glass microspheres with different proportions were used to obtain the different acoustic properties. The mixture consists of E-51 (618) epoxy resin (Shanghai Aotun Chemical Technology Co., Ltd., Shanghai, China), 593 curing agent (Shanghai Aotun Chemical Technology Co., Ltd., Shanghai, China), electronic sealing glue (Changzhou Runxiang Chemical Co., Ltd., Changzhou, China), and a glass microsphere. The composition of the epoxy mixture is shown in Figure 4. The glass microsphere used in the experiment is BR20, with an average diameter of 100 µm and wall thickness between 0.5 and 1 µm, as described in Table 3. To determine the range of the glass microsphere in the mixture, a fabrication testing was conducted. The 24 g epoxy resin, 4 g electronic sealing glue, and 4 g curing agent were mixed. Then, the glass microsphere was added 1 by 1 g to check the viscosity. After the glass microsphere mass exceeded 5 g, the high viscosity made it hard for the mixture to be uniform. So, the glass microsphere masses of 0 g, 1 g, 2 g, 3 g, 4 g, and 5 g, which have a mass ratio of 0%, 3%, 5.9%, 8.5%, 11.1%, and 13.5%, respectively, were prepared to fabricate the piezoelectric composites, as given in Figure 5. The length and width of the composite are 14.6 mm × 14.6 mm with a thickness of 4 mm. The size of the PZT-4 column is 2.1 mm × 2.1 mm. The surface was painted with silver glue (Shenzhen Jingzhe Technology Co., Ltd., Shenzhen, China) to form the electrodes.

The piezoelectric ceramics can be treated as mechanical oscillation systems, which can be characterized by resonant frequency, as given in Equations (A1) and (A2) in Appendix A. The acoustic properties of the 1-3 piezoelectric composite, including acoustic impedance, dielectric constant, and other parameters, were measured by the impedance analyzer PV70A (Beijing Chuangda Electronic Technology Co., Ltd., Beijing, China). To reduce the random error in measurement, each 1-3 piezoelectric composite was measured in ten points, which had five points on both length and width direction. By analyzing the results from the impedance analyzer, the dielectric constant *Ɛ_r_*, the electromechanical coupling coefficient *k_t_*, the acoustic impedance *Z*, the mechanical merit factor *Q_m_*, and the effective electromechanical coupling coefficient *k_eff_* could be obtained. The equations for those parameters are given in Appendix A.

## 3. Results and Discussion

### 3.1. Simulation Results

**Figure 6:**
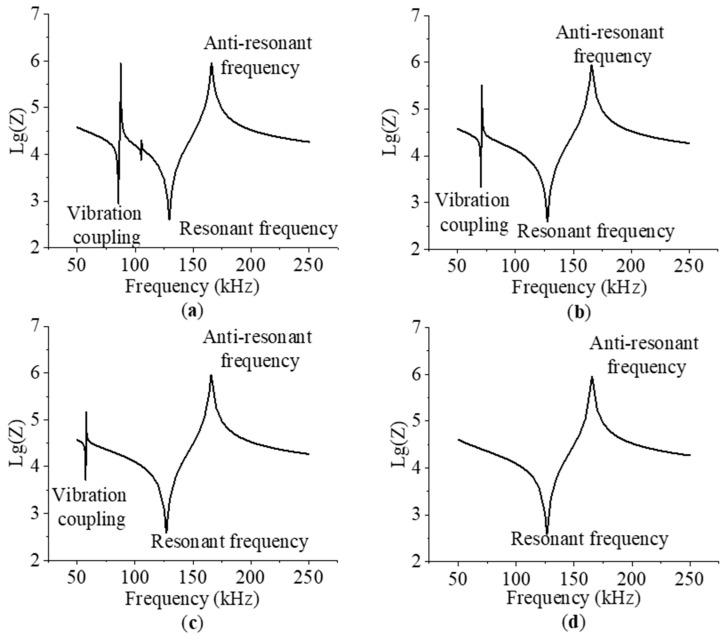
Logarithmic impedance curves of piezoelectric composite with glass microsphere ratio in the following: (**a**) 0%; (**b**) 6%; (**c**) 11%; and (**d**) 14%.

Figure 6 shows the logarithmic impedance curves of 1-3 piezoelectric composites. For the epoxy mixture without a glass microsphere, as given in Figure 6a, the logarithmic impedance curves show two fluctuation points. The first one is in the range of 82–90 kHz, which has an amplitude of about 3. Another has a small fluctuation, which occurs in the range of 102–108 kHz. Figure 6b gives the logarithmic impedance curve of the piezoelectric composite with a 6% glass microsphere. Only one fluctuation point is shown, which is around 68–74 kHz with an amplitude of 2. Compared to the result in Figure 6a, the small fluctuation point disappeared, and the amplitude of the large fluctuation point also decreases by 33%. In addition, the resonant frequency point decreases from 129 to 127 kHz. Figure 6c shows the impedance curve of the piezoelectric composite with an 11% glass microsphere. The resonant frequency decreases to 126 kHz, and the amplitude is about 1.3. The fluctuation point is in the range of 54–60 kHz. Figure 6d gives the impedance curve of the piezoelectric composite with a 14% glass microsphere, which shows a decrease in resonant frequency. No fluctuation is found on the logarithmic impedance curve. From the results above, the glass microsphere added in the epoxy mixture can help to eliminate the coupling effect on 1-3 piezoelectric composites, which reduces the curve fluctuation amplitude until it disappears [15,21].

**Figure 7:**
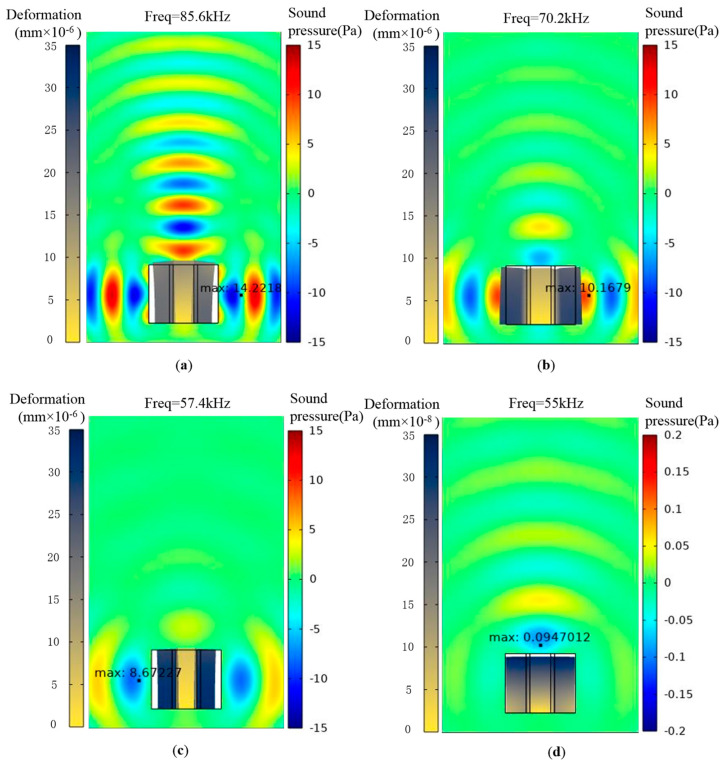
Acoustic wave and composite deformation with glass microsphere ratio in (**a**) 0%, (**b**) 6%, (**c**) 11%, and (**d**) 14%.

Figure 7 shows the sound pressure and composite deformation at the vibration coupling point at different glass microsphere ratios. In Figure 7a, which has no glass microsphere, a lateral acoustic wave is clearly observed. The lateral vibration and deformation of the piezoelectric composite are also observed. The maximum lateral acoustic wave pressure is 14.2 Pa, which is larger than the longitudinal acoustic wave pressure of 12 Pa. The lateral acoustic waves consume a part of the energy, which weakens longitudinal acoustic wave energy, resulting in a decrease in the composite performance. As the mass ratio of the glass microsphere in the epoxy mixture increases to 6%, the lateral acoustic pressure decreases to 10.2 Pa, as given in Figure 7b. As the mass ratio of the glass microsphere continues increasing to 11% and 14%, the lateral sound pressure decreases to 8.6 and 0.1 Pa, respectively. In Figure 7d, the lateral vibrations disappear, and the lateral sound wave is not observed. Due to the adding of the glass microsphere, the frequency in the vibration coupling point is varied.

**Figure 8:**
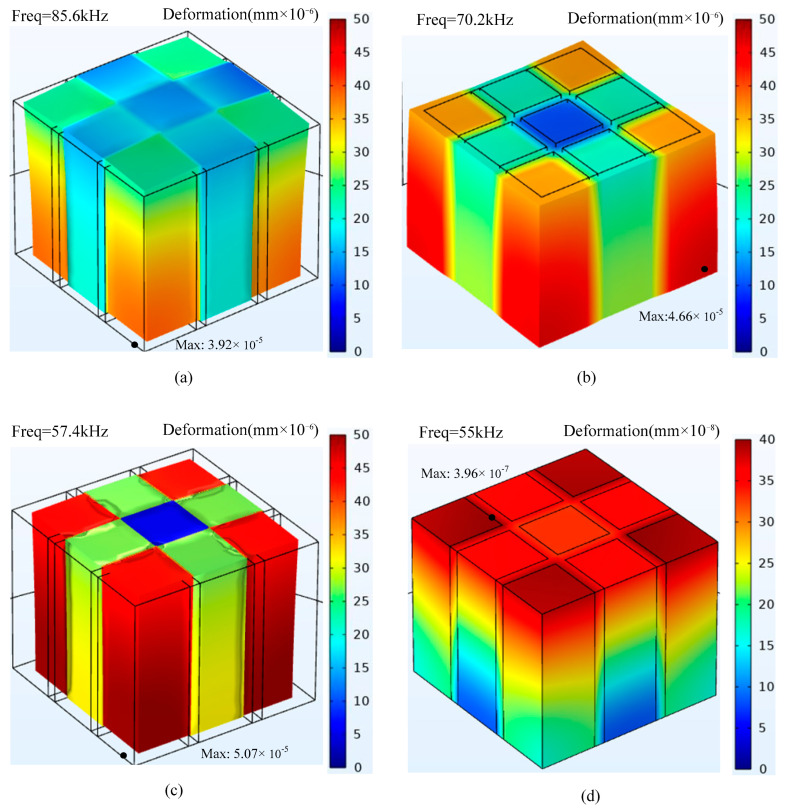
Deformation displacement of piezoelectric composites: (**a**) 0%; (**b**) 6%; (**c**) 11%; and (**d**) 14%.

Figure 8 shows the three-dimensional deformation of piezoelectric composites with different glass microsphere mass ratios. The lateral displacements in piezoelectric composites are 3.9 × 10^−5^ mm, 4.7 × 10^−5^ mm, and 5.1 × 10^−5^ mm for the 0%, 6%, and 11% glass microsphere mass ratio, respectively. In addition, among the piezoelectric columns that undergo deformation, the piezoelectric column located at the four corners has the largest deformation displacement. The piezoelectric column in the center position has the smallest deformation displacement.

### 3.2. Effect of Glass Microsphere on Vibration Coupling

**Figure 9:**
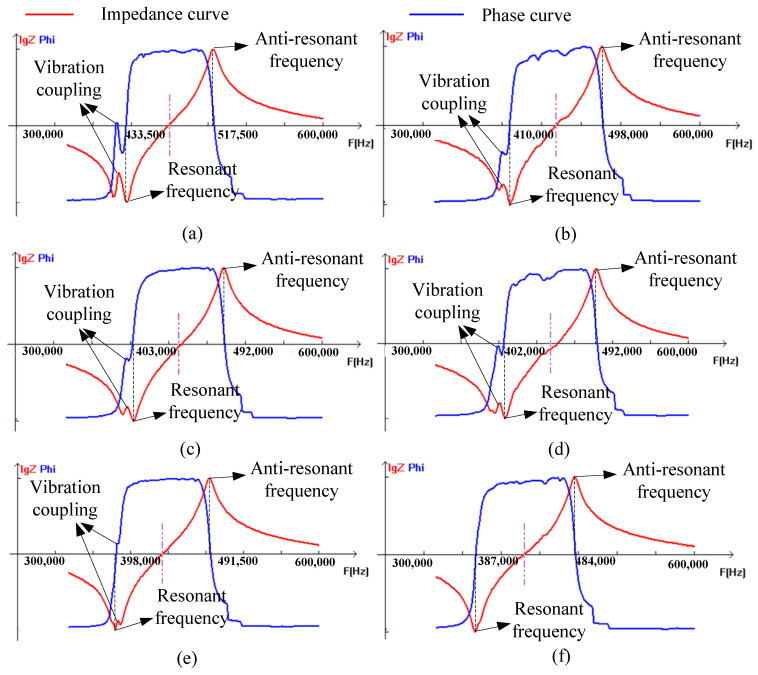
The effect of the glass microsphere mass ratio on vibration coupling: (**a**) 0%; (**b**) 3%; (**c**) 5.9%; (**d**) 8.6%; (**e**) 11.1%; and (**f**) 13.5%.

Figure 9 gives the impedance phase spectrum measured by the piezoelectric composite fabricated in Figure 4. The impedance phase curves at the series resonant frequency have fluctuations with different amplitudes. The vibration is not only produced along the polarization direction of the piezoelectric composite but also the lateral direction. When the vibration frequency at the fluctuations is close to resonant frequency, the coupling vibration is generated, resulting in spurious peaks in the impedance phase curve. Those fluctuations gradually decrease with the increase in the glass microsphere mass ratio of the mixture. As the mass ratio increases to 13.5%, the spurious peaks in the impedance phase curve disappear, indicating that the glass microsphere reduces the coupling vibration in piezoelectric composites. The glass microspheres in the piezoelectric composite are hollow and have greater elasticity than the epoxy resin. The larger the mass ratio of glass microspheres is, the greater the elasticity in the mixture, which can absorb the lateral vibration, ultimately achieving the suppression of vibration coupling. The fluctuation amplitude at a 5.9% mass ratio is lower than that at 8.6%, given in Figure 7c,d, which does not follow the usual tendency, due to fabrication errors.

**Figure 10:**
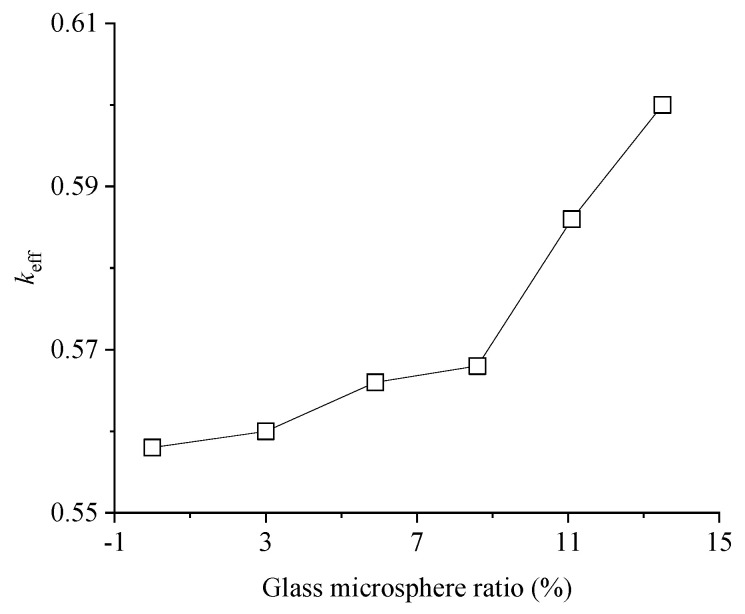
The effect of the glass microsphere ratio on *k_eff_*.

The effective electromechanical coupling coefficient (*k_eff_*) is defined for the non-loss, non-load piezoelectric ultrasonic transducer, which represents the energy utilization rate of the piezoelectric ultrasonic transducer and reflects the relationship between the mechanical energy used to drive the piezoelectric ultrasonic transducer during mechanical resonance and the total energy of the system. Figure 10 shows the effect of glass microspheres on the effective electromechanical coupling coefficient. The elimination of vibration coupling inevitably reduces the energy loss of the piezoelectric composite. *k_eff_* can be used as the indicator for the reduction in the decoupling effect. The increase in *k_eff_* also represents an improvement in the performance of the piezoelectric composite. Figure 9 shows the influence of the glass microsphere mass ratio on *k_eff_*. *k_eff_* increases with the glass microsphere mass ratio increase, which is up to 0.05 as the mass ratio increases from 0 to 13.5%. The vibration coupling effect suppression in a higher glass microsphere mass ratio leads to more energy used for the electromechanical conversion. A smooth slope of *k_eff_* is also observed when the glass microsphere mass ratio is low, which is significantly enhanced in the high mass ratio.

The density of the glass microsphere is 120 kg/m^3^, which is much lower than the epoxy resin density of 1168 kg/m^3^. The glass microsphere shows a trend of floating up in the curing process due to density difference, even if fully stirred and mixed in the fabrication. In the low mass ratio, due to the low viscosity of the mixture, it is easy for the glass microsphere to float up to the upper surface, which is removed after the curing. The actual mass ratio of the glass microsphere is lower than that designed, which leads to a small slope in *k_eff_*. For the high mass ratio of the glass microsphere, the high viscosity can prevent the floating up of the glass microsphere, which makes the mixture more uniform. The effective electromechanical coupling coefficient *k_eff_* is positively correlated with the mass ratio.

The vibration coupling of 1-3 piezoelectric composite materials is mainly caused by the periodic arrangement of the piezoelectric column [22]. To eliminate this interference, the structure of the piezoelectric composite should be modified, such as non-periodic arrangement, non-uniform piezoelectric ceramic column width, through changing the lateral vibration frequency offset from the resonant frequency. However, the non-uniform structure makes the fabrication difficult, which limits the application and development of the 1-3 piezoelectric composite. In this article, changing the filling material property can achieve decoupling, which lays the foundation for the preparation of a high-performance piezoelectric composite.

### 3.3. Effect of Glass Microsphere on Impedance Phase Curve

Figure 9 also shows the impedance and phase curve of the 1-3 piezoelectric composite with different glass microsphere ratios. As the mass ratio of the glass microsphere in the mixture increases, the shape of the impedance curve and phase curve of the 1-3 type piezoelectric composite do not change much. However, the resonant and anti-resonant frequency shift to a low value. The resonant frequency is determined by the effective stiffness of the composite, which is decided by the PZT ceramic and epoxy mixture. Through adding the glass microsphere into the epoxy, the stiffness of the mixture is enhanced, which makes the deformation hard. The vibration of the PZT ceramic is constrained by the mixture, which causes the mixture to deform together. The mixture stiffness increases suppress the PZT ceramic vibration, which decreases the resonant frequency of the 1-3 piezoelectric composite.

### 3.4. Effect of Glass Microsphere on Electrical Parameters

**Figure 11:**
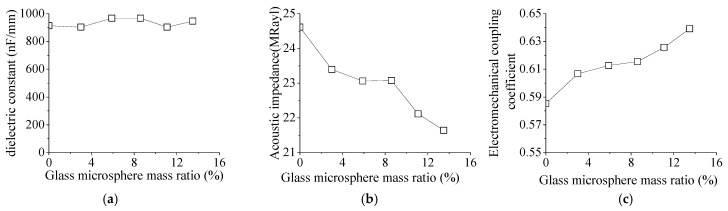
The effect of the glass microsphere mass ratio on the following: (**a**) the dielectric constant; (**b**) acoustic impedance; and (**c**) the electromechanical coupling coefficient.

Figure 11a shows the relationship between the dielectric constant of the piezoelectric composite and the glass microsphere mass ratio. The change in the glass microsphere has little effect on the dielectric constant of the piezoelectric composite. This is because the capacitance, thickness, area, and vacuum dielectric constant of the 1-3 piezoelectric composite are related. However, changing the mass ratio of the glass microsphere does not change those parameters. So, the dielectric constant remains unchanged.

The effect of the glass microsphere mass ratio on the acoustic impedance of the 1-3-type piezoelectric composite is shown in Figure 11b. As the mass ratio of glass microspheres increases, the acoustic impedance of piezoelectric composite material decreases from 24.6 to 21.5 MRayl. The density of glass microspheres is lower than that of epoxy resin. As the mass ratio of glass microspheres increases, the density of the piezoelectric composite decreases. The acoustic impedance of the 1-3 piezoelectric composite is positively correlated with the density and anti-resonant frequencies. Therefore, the acoustic impedance of the 1-3-type piezoelectric composite decreases with the increase in the glass microsphere mass ratio. Acoustic impedance is an indicator of the sound wave propagation resistance. The larger the difference in acoustic impedance between materials is, the greater the propagation loss. The closer the acoustic impedance to the acoustic impedance of the medium is, the better the sound propagation.

Figure 11c shows the effect of different glass microsphere mass ratios on the electromechanical coupling coefficient *k_t_*. As the mass ratio of glass microspheres increases, the electromechanical coupling coefficient of the 1-3 piezoelectric composite gradually increases. The full name of *k_t_* is the thickness expansion and contraction electromechanical coupling coefficient, which reflects the polarization and electrical excitation of the thin sheet along the thickness direction and serves as the electromechanical conversion efficiency parameter of the thickness direction expansion and contraction vibration. Adding glass microspheres to piezoelectric composite materials has a significant decoupling effect, reducing energy consumption. The piezoelectric composite oscillator can be improved. The increase in the thickness electromechanical coupling coefficient enhances the electromechanical conversion ability of composite materials, which improves the transmission and reception performance.

## 4. Conclusions

In this paper, a 1-3 piezoelectric composite model was built to simulate the effects of filling material properties on the performance. Six 1-3 piezoelectric composites were fabricated by changing the glass microsphere mass ratio in the filling material to verify the model. The results indicate that the glass microsphere can help to reduce the Young’s modulus of the filling material, which can suppress the decoupling of the longitudinal and lateral vibration. The glass microspheres can also reduce the series and parallel resonance frequency of the piezoelectric composite. Additionally, the glass microsphere reduces the acoustic impedance of the piezoelectric composite by 12%, increases the electromechanical coupling coefficient by 5.4%, and increases the effective electromechanical coupling coefficient by 8.3%. This study lays the foundation for the high-performance preparation of the PZT-4 composite in the future.

## Figures and Tables

**Table 1:** Material parameter table.

Component Name	Material	Density (kg/m^3^)	Elastic Modulus (Pa)	Poisson’s Ratio
Piezoelectric ceramic column	PZT-4	7500	/	/
Epoxy resin	E51-618	1160	E(T[1/K])	0.38
Glass microsphere	BR20	200		
Air	/	1.205	/	/

**Table 4:** Dicing parameters.

Parameter	Value
Wheel speed (r/min)	2500
Feed rate (mm/min)	5
Kerf space (mm)	2.5
Dicing fluid flow (ml/min)	720
Cooling fluid	Water

**Table 2:** Filling material properties with different glass microsphere ratio.

Parameter	#1	#2	#3	#4
Density (kg/m^3^)	1129	887	744.9	693.9
Young’s modulus (GPa)	1	0.8	0.6	0.4
Poisson ratio	0.38	0.328	0.276	0.25
Glass microsphere ratio (%)	0	6	11	14

**Table 3:** Properties of glass microsphere.

Glass Microsphere Models	Average Diameter d (µm)	True Density ρ_R_ (kg/m^3^)	Bulk Density ρ_B_ (kg/m^3^)	Wall Thickness (µm)	Velocity (m/s)
BR20	100	200	120	0.5–1	2280

## Data Availability

The original contributions presented in the study are included in the article, further inquiries can be directed to the corresponding author.

## Appendix A

Resonant frequency:

(A1) $$f_{r\;}^{1}=f_{r\;}^{0}\cdot \sqrt{\frac{m_{eff}}{m_{eff}+M}},$$

where *m_eff_* is the effective mass of piezoelectric ceramics, which is approximately one-third of the ceramic mass. *M* is the mass of the load, and $f_{r\;}^{0}$ is the resonant frequency of piezoelectric ceramics without load, which can be calculated by the following formula:

(A2) $$f_{r\;}^{0}=\frac{1}{2\pi }\cdot \sqrt{\frac{c_{T}}{m_{eff}}},$$

The dielectric constant represents the piezoelectric composite’s ability to store electrical energy, which is as follows:

(A3) $$\varepsilon_{r}=\frac{cd}{\varepsilon_{0}S},$$

where *c* is the capacitance of the composite, and *d* is the thickness; *ε*_0_ is the vacuum dielectric constant 8.85 × 10^−12^ (F/m), and *S* is the electrode area.

The electromechanical coupling coefficient indicates the conversion efficiency between mechanical energy and electrical energy, which is as follows [23]:

(A4) $$k_{t}=\sqrt{\frac{\pi }{2}\frac{f_{r}}{f_{a}}\tan(\frac{\pi }{2}\frac{f_{a}-f_{r}}{f_{a}})},$$

where *f_r_* and *f_a_* are the resonant frequency and the anti-resonant frequency in thickness mode, respectively.

Acoustic impedance is the propagation resistance of sound waves, which is as follows [24]:

(A5) $$Z=\rho v_{c}=2\rho f_{a}t,$$

where *v_c_* is the sound speed, and *t* is the composite thickness. The density *ρ* is determined by the mass and volume, which can be measured by balance and calculated based on the length, width, and height of composites.

The mechanical quality factor, characterized by the energy consumed by the piezoelectric composite to overcome internal friction during resonance, which is as follows [25]:

(A6) $$Q_{m}=\frac{f_{r}}{f_{+1/2}-f_{-1/2}},$$

where *f_+_*_1/2_ and *f_−_*_1/2_ are frequencies with half-impedance values in *f_r_*.

The effective electromechanical coupling coefficient is the square of the mechanical energy ratio of the stored to total stored energy when the piezoelectric composite is in free oscillation, which can be calculated as follows [26]:

(A7) $$k_{\mathrm{eff}}=\sqrt{\frac{f_{a}^{2}-f_{s}^{2}}{f_{a}^{2}}},$$

For a free piezoelectric oscillation, there are three pairs of frequencies in the admittance circle that exist, which are the resonant frequency *f_r_* and the anti-resonant frequency *f_a_*, the maximum admittance frequency *f_m_* and the minimum admittance frequency *f_n_*, and the series resonance frequency *f_s_* and the parallel resonant frequency *f_p_*. In the case of first-level approximation, they have the relationship below [27]:

(A8) $$\begin{array}{l} f_{p}=f_{a}=f_{n}\\ f_{m}=f_{s}=f_{r}, \end{array}$$

The frequencies of the first and second lateral vibration modes of 1-3 piezoelectric composites are shown as follows [28,29]:

(A9) $$f_{t2}=\frac{v_{t}}{2d},$$

(A10) $$f_{t1}=\frac{v_{t}}{2\sqrt{2}d}$$

where *d* is the spacing of the piezoelectric ceramic column (i.e., the width of the kerf), and *v_t_* is the shear acoustic velocity.
